# Social media as a self-reported source of weight-loss information: a cross-sectional comparison with traditional sources among Arab young adults

**DOI:** 10.3389/fnut.2026.1912985

**Published:** 2026-09-23

**Authors:** Mo’ath F. Bataineh, Rubina Sabir, Ali M. Al-Nawaiseh, Hafiz M. Shahbaz, Amjad H. Jarrar, Habiba I. Ali, Leila Cheikh Ismail, Ayesha S. Al Dhaheri

**Affiliations:** 1 Department of Nutrition and Health, College of Medicine and Health Sciences, United Arab Emirates University, Al Ain, United Arab Emirates; 2 Department of Sport Rehabilitation, College of Sport Sciences, The Hashemite University, Zarqa, Jordan; 3 Department of Clinical Nutrition and Dietetics, College of Health Sciences, University of Sharjah, Sharjah, United Arab Emirates; 4 Nuffield Department of Women’s & Reproductive Health, University of Oxford, Oxford, United Kingdom

**Keywords:** Arab, cross-sectional study, health behavior, nutrition, obesity, social media, weight loss information, young adults

## Abstract

**Background:**

Social media has become an important source of nutrition and weight-loss information among young adults, but concerns persist regarding its quality and reliability, and its influence on weight-loss outcomes remains unclear. This study assessed the prevalence of social media as a weight-loss information source, compared its self-reported outcomes with those of traditional sources such as nutritionists, and examined its association with unhealthy weight-loss behaviors.

**Methods:**

A cross-sectional online survey was conducted using convenience and snowball sampling among Arab young adults residing in the United Arab Emirates. Of 697 respondents, 305 met the inclusion criteria: all were overweight or obese at baseline and reported an intentional weight-loss attempt within the previous 12 months. Participants were categorized by their primary source of weight-loss information; social media, nutritionist, health provider, specialized website, or other sources. Weight-loss success was defined as a ≥5% reduction in initial body weight; unhealthy weight-loss practices were also assessed.

**Results:**

Participants had a mean age of 26.6 ± 6.9 years and a mean body mass index of 31.6 ± 6.0 kg/m^2^; 76.4% were female. Social media was the most reported primary source (39.3%), and 241 participants (79.0%) overall achieved ≥5% weight loss. Weight-loss success was similar between social media users (86.7%) and nutritionist users (82.5%), as was the prevalence of unhealthy weight-loss practices (59.2 and 60.3%, respectively); these descriptive comparisons were not formally tested for statistical equivalence. Younger age, lower educational attainment, and overweight status were significantly associated with high reliance on social media (*p* < 0.05).

**Conclusion:**

Social media is widely used for weight-loss information among young adults and appears associated with self-reported outcomes similar to those of traditional sources such as nutritionists, although the cross-sectional, self-reported design precludes conclusions about comparative effectiveness. Because the sample was restricted to motivated young adults with overweight or obesity, these exploratory, hypothesis-generating findings should be interpreted within this context rather than generalized. They tentatively suggest that public health strategies might enhance the accuracy and credibility of weight-loss content on social media rather than discourage its use, a direction requiring confirmation in studies that directly assess content quality and creator credentials.

## 1 Introduction

In recent years, social media has emerged as a major source of health information, including weight loss, particularly among young adults (1). Over the past two decades, internet users have progressively utilized these platforms to obtain and disseminate health-related information (1). While originally intended for social networking, social media now provides ease of connection with potential social support groups (2) and is recognized as a tool that can enhance communication between individuals and health services (1, 3–6). Adolescents and young adults frequently use social media for information on physical activity, nutrition, and body image (7). A positive association has been observed between social media usage and weight management among adults with obesity (8, 9). These platforms are a popular and preferred source of food and nutrition-related data for weight management (10–13).

Obesity represents a global clinical pandemic with prevalence rates progressively increasing and does not appear to be declining in the near future (14, 15). The economic burden of obesity is substantial, with significant direct and indirect healthcare costs (16, 17). It is associated with numerous comorbidities and health complications (14, 15, 18–20). Literature shows that weight stigma is one of the likely causes of weight gain and related conditions; physiological effects such as social isolation and feelings of rejection are also found to be associated with obesity, creating a cycle that further complicates weight management (21–23). In contrast, cumulative evidence from studies showed that social stigmatization and fat phobia exacerbate weight gain; social support plays an effective role in promoting weight loss among adults with overweight or obesity (23–27).

Recent studies indicate that teenagers and young people tend to rely more on social media due to its accessibility, usability, and popularity. These platforms provide complex health-related ideas in visually appealing, bite-sized content customized to user preferences using algorithms (2). However, concerns have been raised about the suitability and credibility of social media sources regarding health and nutrition related information. Social media influencers greatly play a role in shaping young adults’ nutritional and health perspectives. It has been found in most studies that social media influencers promoted unhealthy diets and unrealistic body images, inaccurate diagnoses and treatments, along with substance use. These social media influencers have been found to play a role in the development of behaviors and habits during the formative growth period, which persist throughout adulthood. These adults were found to have unhealthy weight-loss behaviors such as skipping meals, fasting, misuse of laxatives or diet pills, self-induced vomiting, and extreme caloric restrictions. Previous research has shown that engagement in unhealthy weight control strategies is associated with adverse physical and psychological outcomes, including nutrient deficiencies, disordered eating patterns, and long-term weight cycling. Identifying individuals who rely on such behaviors is therefore important for developing safe and evidence-based weight management interventions (10). Social media contains health-related content that could be used to elevate individuals’ awareness of risky behaviors associated with weight gain and obesity (2). However, some researchers have raised concerns about misinformation, poor quality, and inaccurate nutrition-related content on social media (2, 28). Indeed, most of the food and nutrition-related content on social media has been found to originate from sources other than nutritionists or dietitians (29). Moreover, the most popular accounts on social media do not advertise high-quality nutrition information (30). There is also a concern that social media promotes unhealthy practices that may exacerbate negative and risky health behaviors. This suggests a greater risk of using unhealthy weight loss strategies when counting on social media for weight loss information (31). Consequences of unhealthy weight loss strategies may negate potential weight loss benefits as they have been associated with undesirable nutritional and health outcomes (32).

Social media is widely used for weight management; however, research primarily focuses on its risks, such as misinformation and increased screen time, rather than its role as an accessible source of weight loss information (3). Although the potential of using social media to promote health and weight management is widely recognized (9, 28), information about its use by Arab adults with overweight or obesity for weight management is scarce (33). Limited studies, particularly among Arab young adults, have examined how social media compares to traditional sources like nutritionists and healthcare professionals in terms of effectiveness and associated behaviors (34). This study aims to address this research gap by assessing the prevalence of social media use for weight loss, comparing weight loss success rates across different information sources, and evaluating the link between social media use and unhealthy weight loss practices. The primary outcome was self-reported weight-loss success, defined as a self-reported reduction of at least 5% from the starting body weight, and the primary aim was to compare this outcome between users of social media and users of traditional sources such as nutritionists. Secondary objectives were to estimate the prevalence of each information source, to compare the prevalence of unhealthy weight-loss practices across sources, and to identify the sociodemographic correlates of social media use. Given the cross-sectional and self-reported design, the study was exploratory and hypothesis-generating rather than confirmatory, and we hypothesized that weight-loss outcomes reported by social media users would not differ substantially from those reported by users of traditional sources. Young adulthood (18–39 years) was selected since it is considered a crucial life stage for shaping long-term health behaviors and coincides with the highest use of social media platforms. The study compared weight loss success rate and adoption of unhealthy weight loss practices among individuals who use social media versus those who consult healthcare professionals or nutritionists for weight loss strategies. It is necessary first to establish this comparison to determine the association between growing reliance on social media for weight-loss information and poor health outcomes or risk behaviors, compared with assistance obtained from health professionals.

## 2 Methods

### 2.1 Study design and participants

A cross-sectional study design was used for the collection of retrospective data from individuals who previously tried to lose weight intentionally. A dichotomous item: “In the last 12 months, have you ever tried to lose weight?” was used to assess intentional weight loss attempts. Only participants who responded “yes” to this question completed the full study questionnaire. For recruitment of participants through email, institutional posters, and peer referrals, a convenience and snowball sampling approach was used. The questionnaire was administered online in English and Arabic, and recruitment was based at United Arab Emirates University (Al Ain, United Arab Emirates); all participants were Arab young adults residing in the United Arab Emirates, and eligibility (age, baseline weight status, and an intentional weight-loss attempt) was established from participants’ self-reported responses to the screening items rather than through independent verification. The survey was completed by a total of 697 individuals, of whom 305 (43.8%) met the study’s inclusion criteria and were included in the final analysis. The initial eligible population was defined as respondents who reported an intentional weight-loss attempt in the previous 12 months and satisfied all inclusion and exclusion criteria; the 392 respondents who were not included either answered “no” to the intentional weight-loss screening item or met one or more of the exclusion criteria listed below. Sample size calculation was not performed initially because a non-probability convenience and snowball sampling strategy was used. To maximize statistical power, all eligible respondents recruited during the study period were included. Although a formal *a priori* calculation was not conducted, the achieved sample of 305 participants provides adequate precision for the descriptive estimates; for an observed proportion of 50%, the half-width of the 95% confidence interval (margin of error) is approximately ±5.6%. We nonetheless acknowledge that some information-source subgroups were small, which reduced the precision of subgroup and regression estimates. A 5% threshold was applied because weight loss of this magnitude is generally considered clinically meaningful (35). Inclusion criteria were young adults aged 18 to 39 years who were initially classified as overweight or obese (BMI ≥ 25 kg/m^2^) and had intentionally attempted to lose weight. Exclusion criteria included a baseline (pre-weight-loss) BMI below 25 kg/m2 (i.e., underweight or normal weight), a vegan/vegetarian diet, being pregnant or lactating within the previous year, planning pregnancy, having chronic disease/s, having a diagnosed eating disorder, undergoing bariatric surgery, or experiencing unexplained weight loss. Recruitment occurred between June 4th and July 29th, 2022. These exclusions were applied to reduce confounding of weight-related and dietary behavior: chronic disease, current or recent pregnancy or lactation, and planned pregnancy alter energy requirements, weight trajectory, and dietary management and frequently involve clinically supervised regimens; a vegetarian or vegan diet independently shapes the food-group intake assessed by the food-frequency questionnaire; and unexplained weight loss, a diagnosed eating disorder, and prior bariatric surgery reflect non-intentional or clinically managed weight change rather than the self-directed weight-loss behavior of interest.

### 2.2 Questionnaire

The questionnaire was composed of two main parts. The first part included the Demographic and Cultural Information (DCI) questions, which gathered data on age, gender, weight, height, education level, marital status, health status, smoking status, country, housing, occupation, dieting, main source of weight loss practices, and presence of chronic diseases. The DCI also contained four questions about unhealthy weight loss strategies. The participants were asked to self-report their starting body weight immediately before the start of their attempt to lose weight and their minimum weight during their attempt to lose weight. The percentage of weight loss was calculated as the difference between the starting body weight and the minimum body weight divided by the starting body weight and then multiplied by one hundred.

The recruited participants were classified into two groups based on percentage weight loss from starting body weight. The first group was composed of participants with ≥ 5% weight loss, and the second group had participants with < 5% weight loss. Body Mass Index (BMI) was calculated with the use of self-reported weight (kg) and height (cm). The starting BMI values were used to classify participants into overweight (25 to 29.9 kg/m2), or obese (30 or more kg/m2). Unhealthy weight-loss practices were assessed using four dichotomous items measuring participants’ intentional engagement in specific weight-control behaviors. Participants were asked whether, for more than one day, they had: (1) intentionally restricted food and/or fluid intake (fasting), (2) purposely skipped one or more main meals (breakfast, lunch, or dinner), (3) intentionally omitted an entire food group (i.e., fruits, vegetables, cereals, dairy, or proteins), or (4) intentionally used vomiting, diet pills, supplements, laxatives, diuretics, or other pharmacological agents to induce weight loss. A “yes” response to any item was classified as indicative of engagement in unhealthy weight-loss practices. These four items were adapted from measures used in previous research on unhealthy weight-control behaviors and were selected because of their documented association with adverse health and psychological outcomes (36, 37). The participants were asked to report their main source of information for the weight loss practice/s. Participants selected a single primary (main) source; the questionnaire did not record whether they also drew on additional, secondary sources, and we could not determine whether content encountered on social media had itself been produced by nutritionists or other qualified health professionals. For the final analysis, the responses that reported their major source of information from a physician, or any member of the health team other than a nutritionist (which was considered a category by itself) were reported into one category that was called “health provider,” which included physicians, nurses, and other clinical health team members excluding nutritionists. While responses that reported any of the social media platforms (Facebook, Instagram, etc.) as their major source of information were reported under a single category named “social media.” The responses that reported any of the available health applications as their main source of information for weight loss practice/s were reported together under one category that was named “specialized websites.” For all other reported main sources of information about used weight loss practice/s, such as self, family member, friend, or none disclosed, they were reported under “other sources.”

The second part comprised two validated instruments: a previously validated dietary screening tool was used to develop a 5-item Food Frequency Questionnaire (FFQ) (38) in which participants were asked to report their grains, vegetables, fruits, proteins, and dairy consumption over the preceding week using a four-category response scale (“never,” “1–3 days,” “4–6 days,” and “daily”). For analytical purposes, this ordinal measure was subsequently dichotomized to distinguish between participants who consumed these food groups daily and those who did not. Physical activity was assessed using the short-form International Physical Activity Questionnaire (IPAQ-SF). The tool constitutes seven items designed for the quantification of participants based on engagement in vigorous-intensity physical activity, moderate-intensity physical activity, walking, and sedentary behavior during the preceding seven days. Because both the food-frequency questionnaire and the IPAQ-SF referred to the preceding seven days, they captured recent or habitual intake and activity, which may not coincide with the earlier period during which weight loss was attempted (see Limitations). Both the frequency (days per week) and duration (minutes per day) of participation were reported by the participants for each activity domain. These data were subsequently converted into Metabolic Equivalent Task (MET) minutes per week using established scoring coefficients (8.0 METs for vigorous activity, 4.0 METs for moderate activity, and 3.3 METs for walking). Total weekly MET-minutes were then calculated and used to determine whether participants met recommended physical activity guidelines, with achieving at least 150 min per week of moderate-intensity activity considered indicative of adequate physical activity (39).

### 2.3 Statistical analysis

All statistical analyses were conducted using SPSS version 29.0 (IBM Corp., USA). Data were presented as numbers and percentages for categorical variables, as mean and standard deviation for normally distributed continuous variables and as the median and interquartile range (IQR) for non-normally distributed continuous variables. The normality of all obtained data was tested with the use of the Kolmogorov–Smirnov normality test. The normality test revealed that the weight loss percentage variable was not normally distributed. The nonparametric Kruskal-Wallis H and Mann–Whitney tests were used to compare the effects of independent variables on the weight loss percentage variable. The Chi-square (χ2) test was used to investigate associations between categorical variables. Multiple logistic regression analyses were carried out to detect the impact of the information source for used weight loss practice/s on weight loss status while controlling for confounding factors (age, gender, marital status, education, BMI classification, self-perceived health, employment, meeting PA guidelines, and use of unhealthy weight loss strategies) and their effects on the outcome variable, and the association of sociodemographic factors with the use of social media as a main source of information for weight loss practice/s. Covariates were selected *a priori* on the basis of their established associations with weight-loss outcomes rather than by data-driven selection. We recognize that some of these variables, particularly physical activity and the use of unhealthy weight-loss practices, may lie on the causal pathway between information source and weight loss (i.e., they may act as mediators rather than confounders); the regression models should therefore be interpreted as exploratory and associative. A two-sided *p*-value <0.05 was considered statistically significant. When appropriate, the 95% confidence interval was reported.

## 3 Results

Our study included a total of 305 participants (average age = 26.6 ± 6.9 years; average start BMI = 31.6 ± 6.0 kg/m2; dieting duration = 3.4 ± 3.8 months). Table 1 presents the demographic characteristics of the study participants. Most study participants were female (76.4%). Most of the study participants were 18–24 years old (52.5%), with obesity (54.8%), single (63.3%), holding a bachelor’s degree (60.7%), employed (85.2%), reported good perceived health (42.3%), and obtained diet information from social media (39.3%).

**Table 1:** Socio-demographic characteristics, weight loss behaviors, and diet information source of study participants (*n* = 305).

Variable	*n* (%)
Gender	
Female	233 (76.4)
Male	72 (23.6)
Age category	
18–24	160 (52.5)
25–39	145 (47.5)
Pre-BMI classification	
Overweight	138 (45.2)
Obesity	167 (54.8)
Marital status	
Single	193 (63.3)
Married	95 (31.1)
Divorced/Widow	17 (5.6)
Education level	
School	33 (10.8)
College diploma	27 (8.9)
Bachelor’s degree	185 (60.7)
MSc or PhD	60 (19.7)
Self-perceived health	
Poor/Fair	65 (21.3)
Good	129 (42.3)
Very good/excellent	111 (36.4)
Employment status	
Employed	260 (85.2)
Unemployed	45 (14.8)
Met physical activity guidelines	
Yes	213 (69.8)
No	92 (30.2)
Use of unhealthy weight loss strategies	
Yes	198 (64.9)
No	107 (35.1)
Weight loss practice/s information source	
Other sources	56 (18.4)
Nutritionist	63 (20.7)
Health Specialist	36 (11.8)
Specialized website	30 (9.8)
Social media	120 (39.3)

Figure 1 shows the percentage of participants who attempted to lose weight and managed to lose 5% or more of their initial starting body weight. Most participants (*n* = 241; 79.0%) achieved or exceeded the cutoff value of 5% reduction from the starting body weight.

**Figure 1:**
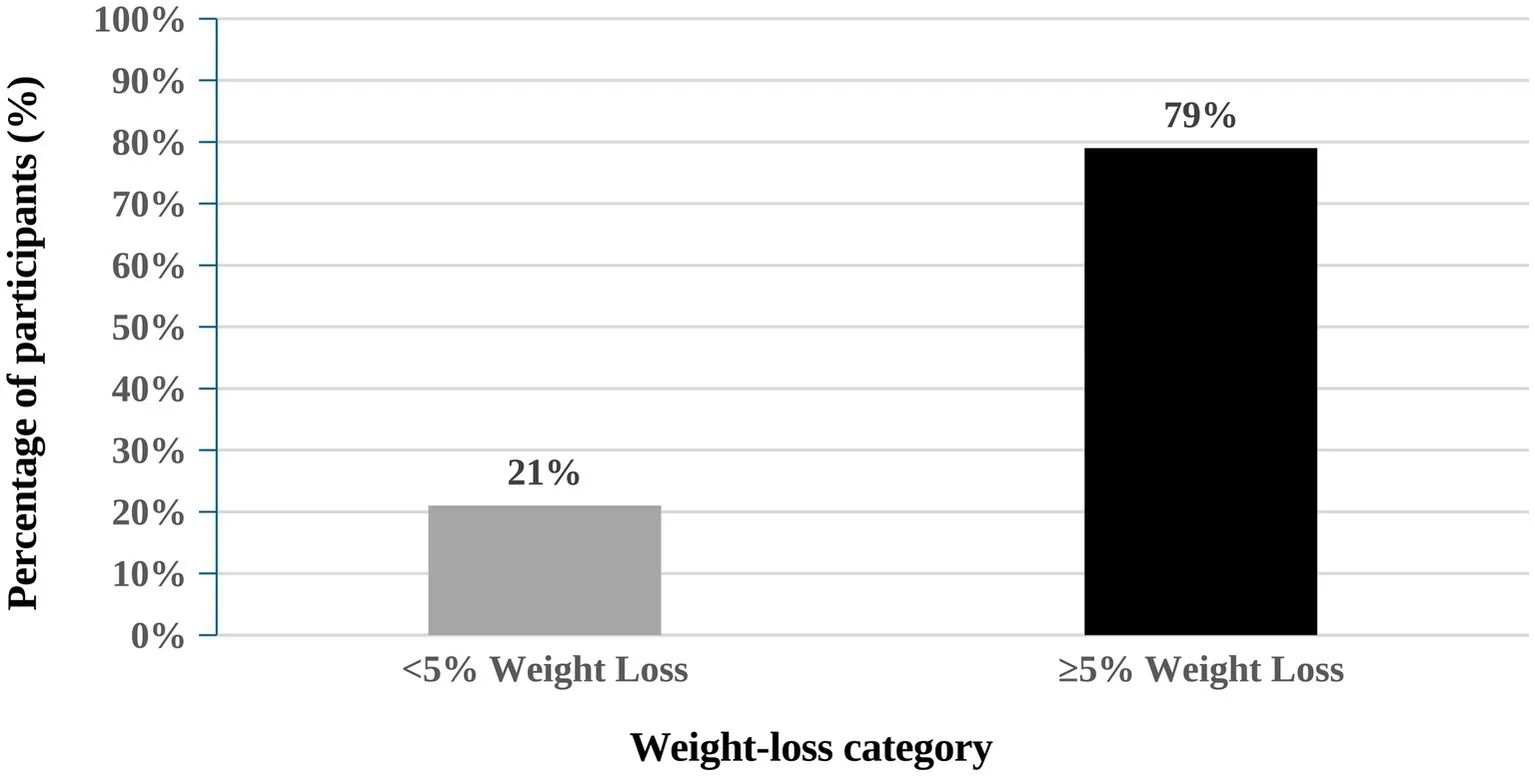
Percentage of participants who achieved the cut-off value (≥5% of starting body weight) for weight loss.

A chi-square analysis was conducted to assess the association between the main source of information for weight loss practices and various demographic characteristics, weight loss, and unhealthy weight loss behaviors (Table 2). A significant association was found between weight loss practices information source and successful weight loss (*p* = 0.002), age category (*p* < 0.001), BMI classification (*p* < 0.001), marital status (*p* = 0.004), education (*p* < 0.001), self-perceived health (*p* = 0.016), meeting physical activity guidelines (*p* < 0.001), and use of unhealthy weight loss strategies (*p* = 0.046). No significant association was detected with gender (*p* = 0.565), or employment status (*p* = 0.202). Rather than pairwise contrasts, these chi-square statistics represent overall (omnibus) associations across the five information-source categories; contribution of groups to each association was evaluated through column percentages. Social media users showed higher weight-loss success and adherence to physical activity.

**Table 2:** Association of demographic characteristics, successful weight loss, physical activity, daily consumption of food groups, and unhealthy weight loss strategies with the main source of information for weight loss practice/s (*n* = 305).

Variable	Other sources	Nutritionist	Health specialist	Specialized website	Social media	X^2^	*p*-value^#^
	*n* (%)	*n* (%)	*n* (%)	*n* (%)	*n* (%)		
Weight Loss (≥5%)						17.085	0.002
Yes	35 (62.5)	52 (82.5)	30 (83.3)	20 (66.7)	104 (86.7)		
No	21 (37.5)	11 (17.5)	6 (16.7)	10 (33.3)	16 (13.3)		
Gender						2.958	0.565
Female	42 (75.5)	51 (81.0)	26 (72.2)	20 (66.7)	94 (78.3)		
Male	14 (25.0)	12 (19.0)	10 (27.8)	10 (33.3)	26 (21.7)		
Age category						31.922	<0.001
18–24	27 (48.2)	21 (33.3)	14 (38.9)	12 (40.0)	86 (71.7)		
25–39	29 (51.8)	42 (66.7)	22 (61.1)	18 (60.0)	34 (28.3)		
BMI classification						21.121	<0.001
Overweight	22 (39.3)	24 (38.1)	10 (27.8)	9 (30.0)	73 (60.8)		
Obesity	34 (60.7)	39 (61.9)	26 (72.2)	21 (70.0)	47 (39.2)		
Marital status						28.816	0.004
Single	36 (64.3)	30 (47.6)	16 (44.4)	19 (63.3)	92 (76.7)		
Married	15 (26.8)	28 (44.4)	17 (47.2)	10 (33.3)	25 (20.8)		
Divorced/Widow	5 (8.9)	5 (7.9)	3 (8.4)	1 (3.3)	3 (2.5)		
Education level						39.564	<0.001
School	2 (3.6)	8 (12.7)	3 (8.3)	4 (13.3)	16 (13.3)		
College diploma	6 (10.7)	2 (3.2)	5 (13.9)	1 (3.3)	13 (10.8)		
Bachelor’s degree	34 (60.7)	31 (49.2)	18 (50.0)	16 (53.3)	86 (71.1)		
MSc or PhD	14 (25.0)	22 (34.9)	10 (27.8)	9 (30.0)	5 (4.2)		
Self-perceived health						18.74	0.016
Poor/Fair	9 (16.1)	12 (19.0)	8 (22.2)	10 (33.3)	26 (21.7)		
Good	27 (48.2)	24 (38.1)	24 (66.7)	11 (36.7)	43 (35.8)		
Very good/excellent	20 (35.7)	27 (42.9)	4 (11.1)	9 (30.0)	51 (42.5)		
Employment status						5.966	0.202
Employed	46 (82.1)	53 (84.1)	27 (75.0)	28 (93.3)	106 (88.3)		
Unemployed	10 (17.9)	10 (15.9)	9 (25.0)	2 (6.7)	14 (11.7)		
Met physical activity guidelines						22.737	<0.001
Yes	37 (66.1)	33 (52.4)	24 (66.7)	18 (60.0)	101 (84.2)		
No	19 (33.9)	30 (47.6)	12 (33.3)	12 (40.0)	19 (15.8)		
Use of unhealthy weight loss strategies						9.681	0.046
Yes	38 (67.9)	38 (60.3)	31 (86.1)	20 (66.7)	71 (59.2)		
No	18 (32.1)	25 (39.7)	5 (13.9)	10 (33.3)	49 (40.8)		

^#^Based on Chi-square analysis, significance *p* < 0.05.

Table 3 shows the associations between the main source of information for weight loss practice/s with the frequency of food group consumption. In general, grains were found to be the major part of participants’ diet, whereas dairy (57.7%), protein (67.9%), fruits (74.8%), and vegetables (64.3%) were not consumed daily by most participants. These percentages represent the proportion of participants who did not report daily consumption of each food group and do not indicate complete avoidance of that group. The chi-square analysis showed no significant differences in the daily consumption of all five food groups in association with the main source of information for weight loss practice/s.

**Table 3:** Daily consumption of food groups according to the main source of information for weight loss practice/s (*n* = 305).

Daily consumption	Total	Other sources	Nutritionist	Health specialist	Specialized website	Social media	X^2^	*p*-value^#^
	*n* (%)	*n* (%)	*n* (%)	*n* (%)	*n* (%)	*n* (%)		
Grains, (7 days/Week)							2.081	0.721
Yes	176 (57.7)	32 (57.1)	37 (58.7)	20 (55.6)	14 (46.7)	73 (60.8)		
No	129 (42.3)	24 (42.9)	26 (41.3)	16 (44.4)	16 (53.3)	47 (39.2)		
Dairy, (7 days/Week)							3.276	0.513
Yes	129 (42.3)	24 (42.9)	26 (41.3)	20 (55.6)	11 (36.7)	48 (40.0)		
No	176 (57.7)	32 (57.1)	37 (58.7)	16 (44.4)	19 (63.3)	72 (60.0)		
Protein, (7 days/Week)							8.462	0.076
Yes	98 (32.1)	13 (23.2)	20 (31.7)	18 (50.0)	7 (23.3)	40 (33.3)		
No	207 (67.9)	43 (76.8)	43 (68.3)	18 (50.0)	23 (76.7)	80 (66.7)		
Fruits, (7 days/Week)							2.99	0.56
Yes	77 (25.2)	11 (19.6)	18 (28.6)	12 (33.3)	6 (20.0)	30 (25.0)		
No	228 (74.8)	45 (80.4)	45 (71.4)	24 (66.7)	24 (80.0)	90 (75.0)		
Vegetables, (7 days/Week)							5.266	0.261
Yes	109 (35.7)	21 (37.5)	23 (36.5)	9 (25.0)	7 (23.3)	49 (40.8)		
No	196 (64.3)	35 (62.5)	40 (63.5)	27 (75.0)	23 (76.7)	71 (59.2)		

^#^Based on Chi-square analysis, significance *p* < 0.05.

Table 4 shows the changes in percentage weight loss according to the main source of information used for weight loss practices, gender, age group, and adherence to physical activity guidelines. A Kruskal-Wallis H analysis revealed significant differences in percentage weight loss based on the information source used [χ^2^(4) = 14.172, *p* = 0.007]. The mean ranks were as follows: 187.75 for health providers, 177.11 for nutritionists, 146.62 for social media, 145.62 for other sources, and 123.24 for specialized websites, corresponding to the group medians (Table 4). Pairwise comparisons indicated no significant difference between “nutritionist” and all other sources of information (*p* > 0.05); the significant pairwise contrasts were between health specialists and both other sources and specialized websites, as indicated by the superscript groupings in Table 4.

**Table 4:** Differences in weight loss percentage according to changes in information source of used weight loss practice/s, gender, and age group (*n* = 305).

Variable	Percentage weight loss Median (IQR)	*p*-value
Weight Loss Practice/s Information Source		0.007^#^
Other Sources	8.8 (3.4–14.0)^a^	
Nutritionist	11.6 (6.8–17.4)^ab^	
Health Specialist	15.5 (6.8–21.3)^b^	
Specialized Website	7.1 (3.5–12.7)^a^	
Social media	10.1 (5.8–17.6)^ab^	
Gender		0.486^¥^
Female	9.5 (5.5–16.7)	
Male	10.5 (5.4–17.9)	
Age category		0.640^¥^
<25	9.6 (5.3–18.0)	
≥25	10.1 (5.6–16.7)	
Met physical activity guidelines		0.049^¥^
Yes	10.4 (5.7–17.3)	
No	9.3 (4.2–15.4)	

IQR, interquartile range. ^#^*p* value based on Kruskal–Wallis H test. ^¥^*p* value based on Mann–Whitney U test. Post-hoc pairwise comparisons were conducted with a Bonferroni correction for multiple comparisons. Median values with different superscript letters are significantly different, and significance values are adjusted for multiple comparisons (*p* < 0.05).

The Mann–Whitney U test showed a significant difference in the reported weight loss percentage based on adherence to physical activity guidelines (*U* = 8,805, *p* = 0.049). In contrast, the Mann–Whitney U test showed no significant differences in the reported weight loss percentage according to gender (*U* = 8,844, *p* = 0.486) or age group (*U* = 11959.5, *p* = 0.640).

Table 5 shows the association of weight loss status with demographics, physical activity status, use of unhealthy weight loss strategies, and the main information source of the used weight loss practice/s. A logistic regression analysis revealed that BMI classification, meeting PA guidelines, and the main source of information for weight loss practice/s were significantly associated with successful weight loss status. Our results showed that odds of achieving weight loss ≥5% were higher relative to reference categories among the individuals who reported greater reliance on social media (OR = 5.91, 95% CI 2.48–14.08) and met physical activity guidelines (OR = 2.05, 95% CI 1.03–4.06), where an odds ratio greater than 1 denotes higher odds of success. In this model, the information-source variable was coded with “other sources” as the reference category, so these odds ratios contrast each source with “other sources” rather than directly comparing social media with nutritionists; the descriptive comparison of social media and nutritionist outcomes (Tables 2, 4) should therefore be read as complementary to, rather than derived from, this model.

**Table 5:** Association of demographics, physical activity, and the main source of information for weight loss practice/s with successful weight loss (*n* = 305).

Variable	Weight loss ≥5%	*p*-value
	Odds ratio (95%CI)	
Gender		0.447
Female	1	
Male	1.364 (0.626–2.895)	
Age category		0.054
<25	1	
≥25	0.436 (0.188–1.015)	
BMI classification		0.017
Overweight	1	
Obesity	2.223 (1.156–4.276)	
Marital status		0.18
Single	1	
Married	0.282 (0.032–2.487)	
Divorced/Widow	0.171 (0.019–1.514)	
Education level		0.299
School	1	
College diploma	0.853 (0.141–5.153)	
Bachelor’s degree	0.375 (0.110–1.282)	
MSc or PhD	0.455 (0.102–2.022)	
Self-perceived health		0.099
Poor/Fair	1	
Good	2.257 (0.999–5.096)	
Very good/excellent	2.243 (0.974–5.164)	
Employment status		0.539
Employed	1	
Unemployed	0.748 (0.296–1.890)	
Met physical activity guidelines		0.04
No	1	
Yes	2.047 (1.033–4.056)	
Use of unhealthy weight loss strategies		0.741
No	1	
Yes	0.894 (0.459–1.739)	
Weight loss practice/s information source		<0.001
Other sources	1	
Nutritionist	3.526 (1.366–9.101)	
Health specialist	3.184 (1.035–9.794)	
Specialized website	1.360 (0.482–3.837)	
Social media	5.913 (2.484–14.075)	

CI, confidence interval.

Table 6 shows the association between sociodemographic factors and the use of social media as the main source of information for weight loss practices. The logistic regression analysis revealed that age, initial BMI, and education level were significantly associated with the reliance on social media for weight loss information. Specifically, younger participants, overweight participants, and individuals with lower levels of education were significantly more likely to use social media as their main source of information for weight loss practices. Our findings showed that older age (≥25 years; OR = 0.46, 95% CI 0.23–0.93) and obesity relative to overweight (OR = 0.35, 95% CI 0.20–0.60) were each associated with lower odds of relying on social media, where an odds ratio below 1 indicates reduced odds.

**Table 6:** Association of sociodemographic factors with the use of social media as the main source of information for weight loss practice/s (*n* = 305).

Variable	Use of social media	*p*-value
	Odds ratio (95%CI)	
Gender		0.706
Female	1	
Male	0.877 (0.443–1.734)	
Age category		0.031
<25	1	
≥25	0.458 (0.225–0.930)	
BMI classification		<0.001
Overweight	1	
Obesity	0.346 (0.201–0.597)	
Marital status		0.941
Single	1	
Married	0.883 (0.428–1.822)	
Divorced/Widow	0.876 (0.195–3.935)	
Education level		0.002
School	1	
College diploma	2.124 (0.673–6.709)	
Bachelor’s degree	1.066 (0.468–2.431)	
MSc or PhD	0.177 (0.048–0.655)	
Self-perceived health		0.188
Poor/Fair	1	
Good	0.555 (0.271–1.133)	
Very good/excellent	0.869 (0.421–1.792)	
Employment status		0.631
Employed	1	
Unemployed	1.210 (0.556–2.631)	

CI, confidence interval.

## 4 Discussion

### 4.1 Principal findings and unique contribution

To the best of our knowledge, this is the first study to provide a balanced comparison of social media against traditional sources for weight loss among Arab-speaking young adults with overweight or obesity. Our key finding is that social media was not only the most prevalent source of weight loss information (39.3%) but was also associated with weight loss success (86.7%) and with a prevalence of unhealthy behaviors similar to that of traditional sources such as nutritionists (a descriptive, rather than statistically tested, equivalence). This challenges the prevailing narrative that frames social media as an inherently risky source of health information and suggests that, in this population of motivated young adults, it functioned as a widely used source associated with self-reported weight-loss success. Our sample exhibited a high overall rate of successful weight loss (79.0%), which likely indicated the enrollment of highly motivated individuals who actively attempted to lose weight. A cautious interpretation of these associations is necessary because our design did not evaluate the healthfulness of the diets followed, whether the content was produced by qualified professionals, the time frame over which weight loss occurred, or whether the losses were maintained.

### 4.2 Comparison with existing literature on effectiveness and usage

The finding that social media was the most cited information source aligns with recent regional and international studies (34, 40). A study conducted in Brazil reported social media as the most common source for weight management among adolescents and adults. It indicated that web-based interventions, which are tailored to individual requirements, are more effective than non-personalized interventions for promoting weight loss and improving dietary habits among individuals with obesity (41). Another study by Waring et al. (12) found that postpartum women preferred a Facebook-delivered intervention, with 70% remaining active through the final intervention module, and 96% of women reported that daily access to the Facebook approach was more convenient than an in-person group. This underscores the perceived accessibility and ease of use of social media. Furthermore, the high rate of successful weight loss (≥5%) among social media users in our study (86.7%) is broadly consistent with a large observational analysis of Reddit weight-loss forums, in which self-reported weight loss of a clinically meaningful magnitude was commonly reported, although that analysis also emphasized that drastic weight loss was rarely attained (42). Other studies have likewise shown the effectiveness of digital and social media-based weight loss interventions (9, 43). Notably, although social media users were the most likely to reach the 5% threshold (Table 5), the greatest median percentage weight loss was recorded among participants who consulted health specialists (15.5%; Table 4), indicating that these two metrics capture different aspects of the weight-loss response. Taken together, literature findings are consistent across diverse social media platforms and population groups, suggesting that social media can potentially play a clinically relevant role in weight loss. However, we cannot establish whether the weight loss was achieved through healthy means or sustained over time, given the cross-sectional and self-reported nature of our data.

### 4.3 The paradox of unhealthy weight loss strategies

A notable and unexpected finding was the lack of significant difference in the prevalence of unhealthy weight loss strategies between users of social media (59.2%) and those advised by nutritionists (60.3%). Rather than indicating that professional guidance fails to protect against risky practices, this similarity may reflect several factors; some health, diet, and sports professionals may, knowingly or unknowingly, encourage strategies targeting rapid or restrictive weight loss, and highly motivated individuals across all sources may adopt such strategies independently of the advice received. Several interpretations are possible. This similarity could reflect a shift in some nutrition practice toward a focus on rapid results, potentially influenced by weight stigma and patient demand for quick fixes (21). Alternatively, it may indicate that the type of structured, and sometimes restrictive, content sought by motivated individuals on social media mirrors the approaches provided by some nutritionists. It is also important to note that the group consulting “health providers” (e.g., physicians) reported the highest rate of unhealthy strategies (86.1%), which may echo reports that some health care providers recommend pharmacotherapy or rapid weight-loss approaches (44). Causal interpretation of these differences is not possible because the study design is cross-sectional and medication use and other clinical details were not measured. These findings may instead reflect the intensity of the weight-loss attempt or the type of professional consulted rather than the information source category itself. Importantly, the similar prevalence of unhealthy practices across sources should not be read as evidence that professional guidance fails to prevent risky behavior. Our composite, four-item indicator classified any endorsed behavior as “unhealthy” and may therefore have captured legitimate, professionally recommended strategies, such as structured meal timing or the temporary reduction of a food group, as unhealthy, which would attenuate any genuine differences between sources.

### 4.4 Demographic correlates of social media use

Our study identified clear demographic patterns in source selection. Younger participants, those with overweight (as opposed to obesity), and individuals with lower educational attainment were significantly more likely to rely on social media. This is a public-health–relevant finding, as it identifies the subgroups most likely to encounter weight-loss information on social media and therefore most likely to benefit from efforts to improve the quality and credibility of that content. This is consistent with research indicating that social media can act as an information equalizer, providing an accessible and approachable source for individuals who might feel excluded from conventional health education channels (12, 41, 45), although we did not collect data on the monetary cost of the information sources used and therefore cannot directly speak to affordability. The dynamic, visual, and peer-driven nature of social media content reduces cognitive barriers to knowledge absorption, making it particularly appealing to younger users (2). On the other hand, older individuals and those with higher education may have greater access to professional services and may be more critical of the credibility of social media content. A cross-sectional web-based study by Ismail et al. (46) conducted in the UAE aligns with our findings, reporting that 80.3% of participants used social media and that such use was associated with adult dietary habits. Most participants reported complete or partial interest (34.0 and 37.0%, respectively) in exploring nutrition-related information on social media, and 41.6 to 52.4% expressed interest in learning about healthy food, recipes, and weight-control or weight-loss diets.

### 4.5 Contrasting findings and limitations in behavioral assessment

In contrast to our findings, Sidani et al. (31) reported, in a nationally representative sample of young adults, greater odds of eating concerns among those with the highest levels of social media use (adjusted odds ratios 2.18, 95% CI 1.50–3.17, and 2.55, 95% CI 1.72–3.78, for the highest quartiles of social media use volume and frequency, respectively). The variation in the results may be due to key methodological differences. Participants in our study were individuals with overweight or obesity who intentionally wanted to lose weight, which may have homogenized their eating behavior regardless of information source, as evidenced by the universal low daily intake of key food groups across all sources. Furthermore, participants’ BMI and weight-loss goals were not reported by Sidani et al. (31). It is also important to acknowledge the limitation of our brief FFQ; future research employing detailed food records is needed to more accurately assess the relationship between information sources and dietary quality. Additionally, an independent association was found between meeting physical activity guidelines and greater weight loss in our data (Table 5), highlighting that physical activity, rather than the information source alone, is an important contributor to weight-loss success and merits fuller integration into digital weight-management strategies.

### 4.6 Limitations and strengths

The current study has several limitations. The cross-sectional design prevents the establishment of cause-effect relationships. The use of self-reported data, including body weight, may introduce bias and limit the generalizability of the results. Furthermore, although our four-item measure of unhealthy weight loss strategies was adapted from instruments used in previous research (36, 37), it did not assess the frequency, duration, or severity of these practices, and compensatory behaviors such as excessive or heat-induced exercise were also not assessed, which could provide a better understanding of these results. We also lacked information on screen time, specific social media platforms used, household income, and potential social isolation effects (47), and we did not analyze the quality or language of the dietary content the participants were exposed to or used. Several other limitations are also present, such as key features of the weight loss process, including the duration of weight loss, maintenance of weight loss, and whether participants were still dieting at the time of the survey, which were not captured. Therefore, the durability of weight loss, or whether it was achieved through healthy means, could not be determined. Intake and physical activity were assessed using the brief five-item food-frequency screener and the IPAQ short form over only the preceding seven days, which may not correspond to the earlier weight-loss period reported over the previous 12 months. These findings were based on self-reported information sources, and we did not verify whether the social media content, specialized websites, or advice from friends and family originated from qualified professionals. Finally, participants’ media and health literacy were also not assessed, which may play a role in shaping both the selection and the interpretation of online weight-loss information; incorporating validated media-literacy measures would be a valuable direction for future research. In addition, our comparisons of weight-loss success and unhealthy-practice prevalence across sources were descriptive; we did not perform equivalence or non-inferiority testing with prespecified margins, so similar percentages and non-significant differences should not be interpreted as statistical evidence that social media is equivalent to nutritionist guidance. The multivariable model also used “other sources” as the reference category and did not directly contrast social media with nutritionists. Accordingly, the study is best regarded as an exploratory, hypothesis-generating analysis of associations between self-reported information sources and recalled weight-loss experiences rather than as evidence of comparable effectiveness or safety.

## 5 Conclusion

This study highlights the widespread use of social media as a primary weight loss information source among young adults, particularly those who are younger and have lower education levels. Our findings suggest that among motivated young adults with overweight or obesity, self-reported weight-loss success and the prevalence of unhealthy weight-loss practices among social media users were comparable to those reported by users of traditional sources such as nutritionists. Given the cross-sectional and self-reported design, these results should be regarded as associative rather than as demonstrating comparable effectiveness. This suggests that social media’s role in weight management is more nuanced than often presumed. Because we did not assess the quality of the content consumed, the platforms used, the intensity of exposure, or the qualifications of content creators, any practice recommendation remains tentative; nevertheless, our findings are consistent with the view that public health efforts might complement, rather than solely discourage, social media use; for example, by collaborating with credible content creators to disseminate evidence-based information, pending confirmation in studies designed to evaluate content quality directly. Simultaneously, enhancing digital health literacy among young adults to critically evaluate weight loss content online is essential. Future research should explore how different types of social media content (e.g., from professionals vs. influencers) influence behaviors to inform these public health strategies.

## Data Availability

The original contributions presented in the study are included in the article/supplementary material, further inquiries can be directed to the corresponding author.
